# Higher cadmium exposure was associated with sex-specific thyroid dysfunction: Consistent evidence from two independent cross-sectional studies based on urinary and blood cadmium measurements

**DOI:** 10.21203/rs.3.rs-3455102/v1

**Published:** 2023-10-19

**Authors:** Ranqi Shao, Liqin Su, Peng Wang, Xu Han, Ting Wang, Jun Dai, Yi Gu, Jiao Luo, Lifang Deng, Jingping Liu

**Affiliations:** National Institute of Environmental Health, Chinese Center for Disease Control and Prevention; National Institute of Environmental Health, Chinese Center for Disease Control and Prevention; Nanjing Agricultural University; National Institute of Environmental Health, Chinese Center for Disease Control and Prevention; National Institute of Environmental Health, Chinese Center for Disease Control and Prevention; Nanjing Agricultural University; Nanjing Agricultural University; National Institute of Environmental Health, Chinese Center for Disease Control and Prevention; Yuhu Center for Disease Control and Prevention; Changde Center for Disease Control and Prevention

**Keywords:** Cadmium, Thyroid dysfunction, Thyroid hormone, Health risk assessment

## Abstract

Population-based studies on the association between cadmium (Cd) exposure and thyroid function are limited and have shown conflicting results. Two independent cross-sectional studies using different Cd biomarkers were carried out in six rural areas with different soil Cd levels in China. Thyroid dysfunction was defined based on levels of thyroid stimulating hormone (TSH) and free thyroxine (FT4). Both multivariable linear regression, multiple logistic regression and restrictive cubic splines models were used to estimate the association between Cd and thyroid dysfunction. For both of the two independent studies, higher Cd levels were observed to be associated with lower TSH levels and higher risk of thyroid dysfunction. The negative relationship between urinary Cd and TSH was found in both total participants (β = −0.072, *p* = 0.008) and males (β = −0.119, *p* = 0.020) but not in females, however, the negative relationship between blood Cd and TSH was only found in females (β = −0.104, *p* = 0.024). Higher urinary Cd (> 2.52 *μ*g/g creatinine) was associated with higher risk of thyroid dysfunction, while higher blood Cd was associated with higher risk of hyperthyroidism status. The adjusted Odds Ratio (OR) for the risk of hyperthyroidism status was 3.48 (95%CI:1.36–8.92) and 6.94 (95%CI:1.23–39.31) times higher with every natural log unit higher in blood Cd in total participants and males, respectively. Results from the two independent cross-sectional studies consistently suggested that higher Cd levels were associated with sex-specific thyroid dysfunction.

## Introduction

Cadmium (Cd) is a widespread environmental contaminant that can spread through soil, water, and air [1, 2]. Agricultural soils have been threatened by Cd from anthropogenic activities in recent years, leading to excessive accumulation of Cd in rice, vegetables and certain foods [3, 4]. Daily dietary intake is the primary route of Cd exposure, especially for non-smokers[3, 5] and there was a close correlation to human body burden [6–8]. Cd can trigger oxidative stress and cause damage to cells, mitochondria, endoplasmic reticulum stress and DNA damage in the human body, potentially leading to the impairment of hypothalamic-pituitary axis, which plays a crucial role in regulating thyroid function [9]. In view of the serious Cd pollution and high prevalence of thyroid dysfunction [10, 11], the association between Cd and thyroid dysfunction need to be explored in more toxicological and population-based studies.

Animal studies have revealed that Cd exposure can cause detrimental effects on thyroid follicular and parafollicular cells [12, 13], inducing hazardous effects on thyroid hormone level abnormalities and thyroid dysfunctions [14, 15]. Moreover, Cd exposure has been found to induce structural damage to the thyroid gland [16] and even increase the risk of developing thyroid cancer [17]. Current evidence has demonstrated that Cd can act as a thyroid disruptor [18].

Human-based studies on the association between Cd exposure and thyroid hormone level and thyroid functions are limited so far, especially no longitudinal study results could be found. Two case-control studies in Iran and Serbia observed higher blood Cd levels in patients with thyroid dysfunction [19, 20]. A cross-sectional study in Japan measured tissue Cd concentrations of inhabitants with a history of living in different Cd-polluted areas and found that Cd concentrations in the post-mortem thyroid gland of individuals residing in Cd-polluted areas and Itai-itai patients were higher than those of individuals residing in non-polluted areas [21]. Moreover, an occupational Cd exposure study in Italy observed negative correlation between urinary Cd levels and free triiodothyronine (FT3) and free thyroxine (FT4) and positive correlation between urinary Cd and thyroid stimulating hormone (TSH) levels [19]. In addition, three studies have revealed a positive association between blood Cd and the risk of hypothyroid status [22–24]. It is of note that no study has explored the association between urinary Cd and thyroid dysfunction, and few studies have verified their conclusions by using both urinary Cd and blood Cd measures which reflect long-term exposure and short-term exposure, respectively [25]. Besides, although age was a significant determinant for Cd levels in a population [26, 27], few studies have focused on older adults.

To comprehensively investigate the association between Cd and thyroid hormone levels and thyroid function status, two independent cross-sectional studies were carried out in six rural areas with different soil Cd pollution levels in China, involving participants across different age stages and measuring different Cd biomarkers.

## Materials and Methods

### Study Design and Participants

Participants in this study were from two independent cross-sectional studies using different Cd biomarkers in six rural areas with different soil Cd levels in China.

The first study using urinary Cd as a biomarker of Cd exposure was carried out in two counties with higher soil Cd levels in 2021. Rural residents aged 18 years or older were recruited if they met the following criteria: (a) had lived in the county for at least ten years and had no language communication problem; (b) consumed local farm products and with no dietary supplement; (c) agreed to complete a face-to-face interview; (d) agreed to participate in the next follow-up. A total of 868 participants were included for analysis after eliminating those missing urinary Cd or thyroid hormone measures.

Another independent study using blood Cd as a biomarker of Cd exposure was carried out in four counties with lower Cd levels from 2017 to 2018. Rural permanent residents aged 65 years or older if they met the following criteria: (a) had lived in the county for at least 30 years and had no language communication problem; (b) consumed local farm products lifelong; (c) agreed to complete a face-to-face interview; (d) agreed to participate in the next follow-up. The details of the study design were described previously [28, 29]. A total of 401 participants with blood Cd thyroid hormone measures were included for statistical analysis.

All participants provided their written informed consent prior to participating in the study. Both of the studies were approved by the Institute for Environmental Health and Related Safety, Chinese Center for Disease Control and Prevention.

### Cd Measurement

Spot urine samples (middle-segment) of at least 50 mL were collected in sterile cups and then handing them involved packing them within 4 hours of collection. Fasting peripheral blood samples were collected in 5 mL purple top (EDTA) vacutainer tubes. All samples were transported to the laboratory with dry ice within 48 hours and stored at −80°C before laboratory analysis.

Urine and blood samples were digested by HNO3 in a microwave digestion device. The urinary Cd and whole blood Cd concentrations were determined by inductively coupled plasma mass spectrometry (ICP-MS, Perkin Elmer NexION 300×, USA). The lowest detection (LOD) for urinary Cd and blood was 0.01 *μ*g/L and 0.03 *μ*g/L, respectively. Laboratory quality control was maintained using certified reference materials and inter-laboratory comparisons. In addition to quality control samples, parallel samples were also determined for quality control. If the deviation was greater than 10%, we repeated the measurement. More details were described elsewhere [30, 31].

### Outcome Assessment

Serum samples were centrifuged at 1500×g for 20 min within four hours. Thyroid measures were measured by immunochemiluminometric assays (ICMA), including thyroid stimulating hormone (TSH), total triiodothyronine (TT3), total thyroxine (TT4) and free triiodothyronine (FT3) and free thyroxine (FT3). The limit of detection (LOD) for TSH, TT3, TT4, FT3 and FT4 was 0.005 mIU/L, 0.1 ng/ml, 0.5 *μ*g/dl, 0.2 pmol/L, and 2.5 pmol/L, respectively.

For males, the reference range of FT4 was 12.8–20.6 pmol/L, and the reference ranges of TSH in two age groups (< 50 years old and ≥ 50 years old) were 0.69–4.66 and 0.64–5.03 mIU/L, respectively. Similarly, for females, the reference range of FT4 was 11.9–18.9 pmol/L, and the reference ranges of TSH in two age groups were 0.74–4.87 and 0.77–5.66 mIU/L, respectively [32, 33].

Thyroid dysfunction was defined as subjects with hyperthyroid status or hypothyroid status. Hyperthyroid status was defined as hyperthyroidism plus subclinical hyperthyroidism. Hyperthyroidism was defined as FT4 concentration above the reference range in combination with TSH concentration below the reference range, whereas subclinical hyperthyroidism was defined as FT4 concentration within the reference range and TSH concentration below the reference range. Hypothyroid status was defined as hypothyroidism plus subclinical hypothyroidism. Hypothyroidism was defined as FT4 concentration below the reference range in combination with TSH concentration above the reference range, whereas subclinical hypothyroidism was defined as FT4 concentration within the reference range and TSH concentration above the reference range.

### Covariates

Information on sociodemographic characteristics, including age and sex, and lifestyle factors, including alcohol consumption, smoking and physical activity were collected from standardized questionnaires. Height and weight were also measured during the interview. Body Mass Index (BMI) was calculated as weight in kilograms divided by height in square meters. Physical activity was classified into the three categories of low, moderate and high according to the Guidelines for Data Processing and Analysis of the International Physical Activity Questionnaire.

### Statistical analysis

We have performed a comprehensive analysis of the data stratified by sex. The Kolmogorov-Smirnov test and P-P plots were used to evaluate whether the data were normally distributed. The categorical variables were presented as percentages, while the continuous variables of normal and skewed distributions were expressed as mean ± standard deviation and median (25% quartile, 75% quartile), respectively. Chi-squared tests, analysis of variance (ANOVA) models and non-parametric tests (Kruskal-Wallis H test) were performed to compare the difference in variables among groups. In linear and logistic regression analyses, whereby urinary Cd and blood Cd, as well as thyroid hormones, were natural log (log) transformed due to their skewed distribution. Furthermore, multivariate linear regression models adjusting for covariations were used to analyze the associations between Cd and thyroid hormones. Multiple logistic regression models adjusting for covariates were used to examine the associations between Cd levels and the risk of thyroid function status. Finally, We used restricted cubic spline (RCS) analysis with four knots at the 5th, 35th, 65th, and 95th percentiles to fit the association between Cd levels and the risk of thyroid dysfunction.

Statistical analyses were performed using R version 4.3.0 (http://www.R-project.org). *p* values of < 0.05 were considered as statistically significant but cautioned against misinterpretations.

## Results

### Urinary Cd and Thyroid Function

#### Description of Participants

The characteristics of participants in higher Cd exposure areas according to sex were presented in Table 1. Among the total 868 participants, there were 325 (37.44%) males and 543 (62.56%) females. The mean ± standard deviation (SD) age for males and females was 63.74 ± 10.48 and 62.45 ± 11.29 years, respectively. The medians (25% quartile value, 75% quartile value) of urinary Cd levels for males and females were 2.316 (1.186, 4.043) *μ*g/g creatinine and 2.717 (1.373, 5.132) *μ*g/g creatinine, respectively (*p* < 0.05).

The males had higher TT3, FT3 and FT4 levels than females. Participants did not differ in age, physical activity and the rates of hyperthyroidism status, hypothyroidism status as well as thyroid dysfunction by sex, but the difference in BMI, alcohol consumption and smoking were statistically significant (*p* < 0.05).

### Urinary Cd and Thyroid Hormone Levels

The adjusted linear regression models between urinary Cd and thyroid hormone levels were shown in Table 2. Log urinary Cd was positively related to Log TT4 [β = 0.042 (0.031, 0.053), *p* < 0.001] and negatively correlated with FT3 [β = −0.031 (−0.038, −0.024), *p* < 0.001], which were not significantly changed after sex stratification. And log TSH levels decreased with increasing log urinary Cd levels in the total participants [β = −0.072 (−0.099, −0.045), *p* = 0.008] and the males [β = −0.119 (−0.170, −0.068), *p* = 0.020], but the correlation was not significant in females.

### Urinary Cd and Thyroid Dysfunction

**Fig.1:**
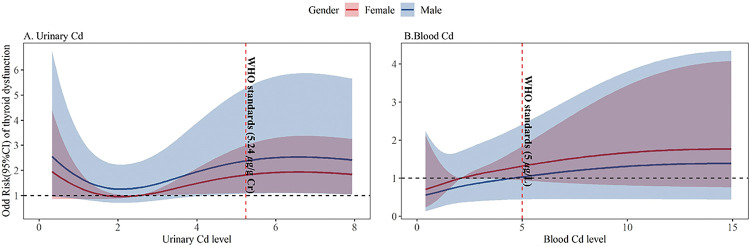
RCS analysis on the association between Cd exposure and the risk of thyroid dysfunction.

The multiple logistic regression results between urinary Cd and the prevalence risk of thyroid dysfunction were presented in Table 3. With every log unit higher in urinary Cd, the adjusted OR for the prevalence risk of hyperthyroidism status and hypothyroidism status was 1.66(0.62,4.47) and 1.31(0.77, 2.22) times higher in total participants, respectively. Besides, a non-linear association between urinary Cd and thyroid dysfunction was observed from RCS analysis with the cut-off points of 1.69 *μ*g/g creatinine and 2.52 *μ*g/g creatinine, as shown in Fig. 1. Sex-specific difference was observed in the association between urinary Cd and the risk of thyroid dysfunction, respectively, as shown in Fig. 1. When the urinary Cd higher than 2.52 *μ*g/g creatinine, males had higher risk of thyroid dysfunction than females.

### Blood Cd and Thyroid Function

#### Description of Participants

The characteristics of participants in relatively low Cd areas according to sex were shown in Table 4. Among the total 401 participants, there were 148 (36.91%) males and 253 (63.09%) females. The mean ± SD age for males and females was 73.75 ± 4.70 and 73.95 ± 5.66, respectively. The medians (25% quartile value, 75% quartile value) of whole blood Cd levels for males and females were 3.136 (1.773, 6.747) *μ*g/L and 1.765 (1.260, 3.943) *μ*g/L, respectively (*p* < 0.05).

And the males had higher FT3, lower TT4 levels and lower risk of hyperthyroid status than females. Participants did not differ in age, physical activity as well as the risk of hypothyroid status and thyroid dysfunction by sex, but the difference in BMI, alcohol consumption and smoking were statistically significant (*p* < 0.05).

### Blood Cd and Thyroid Hormone Levels

The adjusted linear regression models between urinary Cd and thyroid hormone levels were shown in Table 5. Log FT4 increased with increasing Log blood Cd [β = 0.034 (0.027, 0.041), *p* < 0.001], which were not significantly changed after sex stratification, while the positive relation between log blood Cd and lg TT3 levels was only found in total participants and females. In females, log TSH levels were negatively associated with log blood Cd levels [β = −0.104 (−0.150, −0.058), *p* = 0.024] and log FT4 levels were positively associated with log blood Cd levels [β = 0.030 (0.018, 0.042), *p* = 0.013] while the negative association between blood Cd and TT4 was found in males [β = −0.040 (−0.058, −0.022), *p* = 0.028].

### Blood Cd and Thyroid Dysfunction

Results from RCS analysis have also revealed that higher blood Cd was associated with higher risk of dysfunction (Fig. 1). The adjusted logistic regression results between high Blood Cd and the prevalence risk of thyroid dysfunction were presented in Table 6. The adjusted OR(95%CI) for the risk of subclinical hyperthyroidism and hyperthyroidism status was 3.92 (1.29, 11.93) and 3.48 (1.36, 8.92) times higher with every natural log unit higher in blood Cd in total participants. The positive association between blood Cd and hyperthyroidism status was observed in males (OR = 6.94, *p* = 0.028), while the association had no statistical significance in females.

## Discussion

Our two independent cross-sectional studies consistently showed that higher Cd was associated with lower TSH level, higher FT4 level and higher risk of thyroid dysfunction. The negative relationship between urinary Cd and TSH was significant in both total participants and males but not in females, however, the negative relationship between blood Cd and TSH was only significant in females. Higher urinary Cd was associated with higher prevalence of thyroid dysfunction, while higher blood Cd was associated with higher risk of hyperthyroidism status and thyroid dysfunction. Our results consistently suggested that higher Cd was associated with higher risk of sex-specific thyroid dysfunction.

Several previous studies supported our findings on the association between Cd exposure and thyroid hormone levels. Results from the National Health and Nutrition Examination Survey data (NHANES 2007–2008) showed that Cd measured in both blood and urine was associated with lower TSH [35], and the association was observed in both adults and adolescents [36]. A negative association between Cd in cord blood and TSH in neonatal blood was found in a Tokyo cross-sectional study [37]. On the other hand, the positive association between blood Cd and FT4 shown in our study could also be found in the US NHANES (2007–2008 and 2007–2010) and Korea National Health and Nutrition Examination Survey (KNHANES 2013) [24, 36]. Additionally, two occupational studies consistently showed a negative association between urinary Cd and FT3 [38, 39], while a positive association between urinary Cd and TT4 was seen in a Sweden study involving 548 pregnancies [40].

However, there were also some studies that did not support our results. Positive association between urinary Cd and TSH was shown in two occupational studies [38, 39], and no significant association between blood Cd and TSH was observed in cross-sectional studies from America and China [23, 41]. In addition, a negative association between blood Cd and FT4 was observed in a cross-sectional study involving 5628 Chinese adults [23], which was opposite to the results in our study. Other inconsistent results could also be observed in the studies on Chinese children [40] and US adults and adolescents [36]. It is worth noting that the average age of our participants was much older than participants in other studies, therefore, the inconsistent results could partly be explained by the difference in age and gender. Moreover, the non-linear dose-response relationship between Cd and thyroid hormone levels shown in our study and some other studies could explain more [18, 40].

As far as we know, only three previous studies have investigated the relationship between blood Cd exposure and thyroid function, two studies in China [22, 23] and one study in Korea [24] consistently indicated that higher blood Cd was associated with higher risk of hypothyroidism, which was consistent with our findings in the study measuring urinary Cd concentrations (OR = 1.31). However, no significant association between blood Cd and hypothyroidism was observed in our study measuring blood Cd concentrations. Meanwhile, blood Cd was associated with higher risk of hyperthyroidism status was observed in our study, while no significant association was observed in the 2014 SPECT-China study involving 5628 Chinese adults [23]. It is worth noting that the blood Cd level of our participants was higher than participants in three previous studies, which may partly explain the discrepancy in study results. Moreover, the inconsistent results from different studies may be due to the age and gender distribution of the study participants, sample size and the selection of biomarkers of Cd, longitudinal studies are needed to verify the association between Cd and thyroid dysfunction in the future.

The sex-specific difference in the association between Cd and thyroid function was observed in our study, which could also be found in some previous studies. Females with higher blood Cd were associated with higher risk of hypothyroid status were observed in the 2014 SPECT-China study [23], inversely, the KNHANES 2013 [24] showed that the association was only observed in males but not in females. Meanwhile, the sex-difference in the association between Cd and thyroid hormone levels was observed in two US cross-sectional studies (NHANES 2007–2010 and NHANES 2011–2012). The sex-specific response to Cd exposure might be explained by the physiological difference and the difference in smoking rates, diet, physical activity and so on. More evidence could be found in experimental studies on other health effects of Cd exposure, such as glucose homeostasis [42] and neurotoxicity [43], suggesting the sex-specific effect of Cd exposure need to be emphasized in the future.

Although the mechanism of Cd-induced thyrotoxicity has not yet been fully understood, animal studies have revealed that Cd exposure can act as a thyroid disruptor. Previous studies demonstrated that Cd exposure in animals is associated with the trend of hypothyroxinemia [14, 18]. Meanwhile, investigations in lower vertebrates reported that higher Cd could induce thyroid structural injuries and thyroid signaling disruption of bufo gargarizans and these studies indicated that high-dose Cd cause other adverse outcomes by disrupting the thyroid system [13, 44]. Furthermore, the mechanism of Cd-induced thyrotoxicity was regarded as the consequence of Cd effects on pituitary secretion in some in vivo studies on rats [45–47]. Additionally, a mice model showed that Cd exposure could facilitate thyroid follicular cell pyroptosis by inhibiting Nrf2/Keap1 signaling, thereby disrupting thyroid tissue structure and endocrine function, which offers novel insights into the Cd-mediated detrimental consequences on thyroid homeostasis [16].

Our study has some strengths. Firstly, our study examined the associations between Cd status and FT3 and FT4, which were seldom measured in prior studies. Secondly, we evaluated Cd status using both urinary Cd and blood Cd measures in two independent studies, allowing for both short-term and long-term Cd status assessments and enabling comparison of study results with other studies. In addition, the Reference Intervals of thyroid hormones in our study considered the gender and age difference of Chinese people, which made the classification of thyroid dysfunctions more reliable.

There are also some limitations. Firstly, the research only assessed a signal time point and lacked longitudinal data, thereby the observed association could not indicate causation. Secondly, hyperthyroidism and hypothyroidism were defined based on fasting serum thyroid hormone levels only, the lack of clinical diagnosis could induce misclassification bias to some extent.

## Conclusion

Our two independent cross-sectional studies consistently showed that higher Cd was associated with lower TSH level, higher FT4 level and higher risk of thyroid dysfunction, and the associations were sex-specific. Further well-designed longitudinal studies are required to confirm our findings.

## Figures and Tables

**Table 1:** Characteristics of the 868 participants in higher Cd exposure areas measuring urinary Cd.

Characteristics	Total(n = 868)	Male(n = 325)	Female (n = 543)	*p*-value
Age(years)	62.93 ± 11.01	63.74 ± 10.48	62.45 ± 11.29	0.096
BMI(kg/m^2^)	23.97 ± 3.57	23.45 ± 3.38	24.29 ± 3.64	0.001*
Waist-hip ratio	0.90 ± 0.08	0.90 ± 0.07	0.89 ± 0.08	0.069
Smoker (%)	30.20%	71.40%	5.50%	< 0.001*
Alcohol consumer (%)	24.20%	50.80%	8.30%	< 0.001*
Physical activity class				0.955
1(%)	3.20%	3.10%	3.30%	
2(%)	2.30%	2.50%	2.20%	
3(%)	94.50%	94.50%	94.50%	
Urinary Cd level (*μ*g/g creatinine)	2.519(1.324,4.658)	2.316(1.186,4.043)	2.717(1.373,5.132)	< 0.001*
TSH (mIU/L)	2.610(1.818,3.845)	2.370(1.670,3.540)	2.740(1.915,4.035)	0.861
TT3 (nmol/L)	1.771(1.602,1.925)	1.848(1.663,2.002)	1.725(1.571,1.894)	< 0.001*
TT4 (nmol/L)	98.198(87.870,109.56)	98.842(88.546,110.167)	97.941(87.645,109.395)	0.142
FT3 (pmol/L)	6.853(6.36,7.377)	7.146(6.530,7.654)	6.714(6.299,7.207)	< 0.001*
FT4 (pmol/L)	14.157(12.999,15.573)	14.672(13.514,16.345)	13.9(12.87,15.187)	< 0.001*
Hyperthyroidism status (%)	3.0%	2.5%	3.3%	0.310
Hypothyroidism status (%)	9.9%	10.8%	9.4%	0.293
Thyroid dysfunction (%)	12.9%	13.2%	12.7%	0.450

Normally and non-normally distributed continuous variables were expressed as the Mean ± SD and the median (25% quartile value, 75% quartile value), respectively. Categorical variables are

presented as percentages.

* *P* < 0.05

**Table 2:** Adjusted linear regression models between Urinary Cd and thyroid hormone levels.

	Total(n = 868)		Male(n = 325)		Female(n = 543)	
	β(95%CI)	*p*-value	β(95%CI)	*p*-value	β(95%CI)	*p*-value
Log-TSH	−0.072(−0.099,−0.045)	0.008*	−0.119(−0.170,−0.068)	0.020*	−0.055(−0.087,−0.023)	0.081
log-TT3	−0.009(−0.015,−0.003)	0.180	−0.005(−0.018,0.008)	0.709	−0.009(−0.016,−0.002)	0.204
log-TT4	0.042(0.031,0.053)	<0.001*	0.054(0.031,0.077)	0.017*	0.037(0.025,0.049)	0.003*
log-FT3	−0.031(−0.038,−0.024)	<0.001*	−0.036(−0.049,−0.023)	0.007*	−0.028(−0.036,−0.020)	<0.001*
log-FT4	0.002(−0.007,0.011)	0.853	−0.003(−0.023,0.017)	0.884	0.004(−0.006,0.014)	0.686

Adjusted for age, BMI, Waist-hip ratio, smoking, alcohol consumption and physical activity.

* *P* < 0.05

**Table 3:** Logistic regression models between high Urinary Cd and the risk of Thyroid dysfunction.

	Total		Male		Female	
Characteristics	No. of event/No. of subjects	Adjusted OR (95% CI)	No. of event/No. of subjects	Adj usted OR [95% CI)	No. of event/No. of subjects	Adj usted OR [95% CI)

Hyperthyroidism status						
Low Cd group ^a^	20/68	1.00(reference)	7/277	1.00(reference)	13/412	1.00(reference)
High Cd group ^b^	6/179	0.87(0.33, 2.30)	1/48	0.22(0.02, 2.92)	5/131	1.15(0.39, 3.43)
Hypothyroidism status						
Low Cd group ^a^	61/689	1.00(reference)	27/277	1.00(reference)	34/412	1.00(reference)
High Cd group ^b^	25/179	1.77(1.05, 2.98)*	8/84	1.99(0.78, 5.13)	17/131	1.75(0.92, 3.32)
Thyroid dysfunction						
Low Cd group ^a^	81/689	1.00(reference)	34/277	1.00(reference)	47/412	1.00(reference)
High Cd group ^b^	31/179	1.54(0.96, 2.47)	9/48	1.47(0.60, 3.59)	22/131	1.60(0.91, 2.83)

Adjusted for age, BMI. smoking, alcohol consumption and physical activity.

a Low Cd group was defined as < 5.24 *μ*g/g creatinine

b High Cd group was defined as ≧5.24 *μ*g/g creatinine

* *P* < 0.05

**Table 4:** Characteristics of the 401 participants in lower Cd exposure areas measuring blood Cd.

Characteristics	Total (n = 401)	Male(n = 148)	Female (n = 253)	*p*-value
Age(years)	73.88 ± 5.32	73.75 ± 4.70	73.95 ± 5.66	0.719
BMI(kg/m2)	23.51 ± 3.51	22.79 ± 3.20	23.93 ± 3.63	0.002*
Smoker (%)	35.40%	76.40%	11.50%	<0.001*
Alcohol consumer (%)	34.20%	66.20%	15.40%	<0.001*
Physical activity class				0.447
1(%)	3.50%	2.00%	4.30%	
2(%)	13.50%	15.50%	12.30%	
3(%)	83.00%	82.40%	83.40%	
Blood Cd level (*μ*g/L)	2.085(1.402,4.801)	3.136(1.773,6.747)	1.765(1.260,3.943)	0.004*
TSH (mIU/L)	1.750(1.120,2.830)	1.610(1.112,2.615)	1.780(1.120,2.970)	0.713
TT3 (nmol/L)	1.850(1.600,2.070)	1.810(1.545,1.990)	1.870(1.630,2.080)	0.075
TT4 (nmol/L)	115.300(102.000,129.000)	113.25(98.175,123.575)	116.200(103.800,130.500)	0.004*
FT3 (pmol/L)	4.600(4.230,4.900)	4.630(4.240,5.033)	4.580(4.220,4.850)	0.046*
FT4 (pmol/L)	16.100(14.700,17.400)	16.200(15.100,17.800)	15.900(14.600,17.300)	0.071
Hyperthyroidism status (%)	10.0%	5.4%	12.6%	0.013*
Hypothyroidism status (%)	6.2%	8.8%	4.7%	0.082
Thyroid dysfunction (%)	16.2%	14.2%	17.3%	0.244

Normally and non-normally distributed continuous variables were expressed as the Mean ± SD and the median (25% quartile value, 75% quartile value), respectively. Categorical variables are

presented as percentages.

* *P* < 0.05

**Table 5:** Adjusted linear regression models between log Blood Cd and thyroid hormone levels.

	Total(n = 401)		Male(n = 148)		Female(n = 253)	
	β(95%CI)	*p*-value	β(95%CI)	*p*-value	β(95%CI)	*p*-value
log-TSH	−0.059(−0.094, −0.024)	0.088	−0.041(−0.095, 0.013)	0.445	−0.104(−0.150, −0.058)	0.024*
log-TT3	0.020(0.013, 0.028)	0.009*	0.0220(0.010, 0.034)	0.064	0.021(0.011, 0.032)	0.048*
log-TT4	−0.012(−0.024, 0)	0.329	−0.040(−0.058, −0.022)	0.028*	0.022(0.005, 0.039)	0.198
log-FT3	0.034(0.027, 0.041)	< 0.001*	0.043(0.032, 0.054)	< 0.001*	0.026(0.017, 0.035)	0.006*
log-FT4	0.019(0.01, 0.028)	0.029*	0.010(−0.002, 0.022)	0.420	0.030(0.018, 0.042)	0.013*

Adjusted for age, BMI, Waist-hip ratio, smoking, alcohol consumption and physical activity.

* *P* < 0.05

**Table 6:** Logistic regression models between high Blood Cd and the risk of Thyroid dysfunction.

	Total		Male		Female	
Characteristics	NE/NS	Adjusted OR (55% CI)	NE/NS	Adjusted OR (95% CI)	NE/NS	Adjusted OR (95% CI)

Hyperthyroidism status						
Low Cd group ^a^	26/305	1.00(reference)	4/102	1.00(reference)	22/203	1.00(reference)
High Cd group ^b^	14/95	2.06(0.97, 4.37)	4/46	2.46(0.56, 10.33)	10/50	2.12(0.36, 5.2)
Hypothyroidism status						
Low Cd group ^a^	17/305	1.00(reference)	8/102	1.00(reference)	9/203	1.00(reference)
High Cd group ^b^	8/96	1.57(0.62, 3.95)	5/46	1.53(0.45, 5.54)	3/50	1.42(0.32, 6.26)
Thyroid dysfunction						
Low Cd group ^a^	43/305	1.00(reference)	12/102	1.00(reference)	31/203	1.00(reference)
High Cd group ^b^	22/96	1.95(1.07,3.57)*	9/46	1.93(0.71, 5.25)	13/50	1.93(0.37, 4.26)

Adjusted for age, BMI. smoking, alcohol consumption and physical activity.

a Low Cd group was defined as < 5 *μ*g/L

b High Cd group was defined as ≧5 *μ*g/L

* *P* < 0.05
